# Editorial: Theoretical advances and practical applications of spiking neural networks

**DOI:** 10.3389/fnins.2024.1406502

**Published:** 2024-04-22

**Authors:** Gaetano Di Caterina, Malu Zhang, Jundong Liu

**Affiliations:** ^1^Neuromorphic Sensor Signal Processing Lab, Department of Electronic and Electrical Engineering, Centre for Image and Signal Processing, University of Strathclyde, Glasgow, United Kingdom; ^2^Centre for Future Media, School of Computer Science and Engineering, University of Electronic Science and Technology of China, Chengdu, Sichuan, China; ^3^Learning and Intelligent Systems Lab, School of Electrical Engineering and Computer Science, Ohio University, Athens, OH, United States

**Keywords:** Spiking Neural Networks (SNN), Neuromorphic Engineering (NE), event-based sensing, neural networks, artificial intelligence

## 1 Introduction

Neuromorphic engineering has experienced a significant growth in popularity over the last 10 years, going from being a niche academic research area, often confused with deep learning and mostly unknown to the wider industrial community, to being the main focus of many funding calls, significant industrial endeavours, and national and international initiatives. The advent to market of neuromorphic sensors, with a related widening understanding of the event-based sensing paradigm, combined with the development of the first neuromorphic processors, has steered the wider academic community and industry toward the investigation and use of Spiking Neural Networks (SNN). Very often overlooked in favour of the now extremely popular Deep Neural Networks (DNN), SNNs have become a serious alternative to DNNs, in application domains where size, weight and power are key limiting factors to the deployment of AI systems, such in Space applications, Security and Defence, Automotive, and more generally AI at the Edge. Nonetheless, there are many aspects of SNNs that still require significant investigation, as there are many unexplored avenues in this regard. To this aim, the articles accepted in this special topic present novel research works that focus on methodologies for training of SNNs and on the use of SNN in real life applications.

## 2 About the papers

The articles published in this special topic cover a varied set of application domains, including image processing (such as denoising and segmentation), analysis of biosignals (for seizure detection), activity recognition through wearable devices, and audio processing for the cocktail party effect. Moreover, the topic hosts also two papers on novel learning methods, such as meta-spikepropamine, and the chip-in-loop SNN proxy learning.

In the article “*Efficient and generalizable cross-patient epileptic seizure detection through a spiking neural network*”, Zhang et al. propose an EEG-based spiking neural network (EESNN) with a recurrent spiking convolution structure, leveraging the biological plausibility of SNNs, to take better advantage of temporal and biological characteristics of EEG signals.

Li Y. et al. propose the application of the SNNs to time series of binary spikes generated through wearable devices, in their work entitled “*Efficient human activity recognition with spatio-temporal spiking neural networks*”. The results reported indicate that SNNs achieve competitive accuracy while significantly reducing energy in this application.

In “*Explaining cocktail party effect and McGurk effect with a spiking neural network improved by Motif-topology*” Jia et al. introduce a novel Motif-topology improved SNN (M-SNN) to enhance the network's ability to tackle complex cognitive tasks. The experimental results show a lower computational cost and higher accuracy and a better explanation of some key phenomena of these two effects, such as new concept generation and anti-background noise.

In their article “*Meta-SpikePropamine: learning to learn with synaptic plasticity in spiking neural networks*”, Schmidgall et al. propose a novel bi-level optimization framework that integrates neuroscience principles into SNNs to enhance online learning capabilities. The experimental outcomes underscore the potential of neuroscience-inspired models to advance the field of online learning, marking a promising direction for future research.

Li X. et al. propose a biologically-plausible algorithm named the mixture of personality (MoP) improved spiking actor network (SAN), in their work “*Mixture of personality improved spiking actor network for efficient multi-agent cooperation*”. The experimental results on the benchmark cooperative overcooked task show that the proposed MoP-SAN algorithm could achieve higher performance for the paradigms with (learning) and without (generalization) unseen partners.

In the article “*SPIDEN: Deep Spiking Neural Networks for Efficient Image Denoising*” Castagnetti et al. explore the use of SNNs for image denoising applications, with the goal of reaching the accuracy of conventional Deep Convolutional Neural Networks (DCNNs), while reducing computational costs. The authors present a formal analysis of data flow through Integrate and Fire (IF) spiking neurons, and establish the trade-off between conversion error and activation sparsity in SNNs. Experimental results demonstrate that the design goals are effectively achieved.

Yue et al. propose a novel three-stage SNN training scheme for segmenting human brain images, in the work “*Spiking Neural Networks Fine-Tuning for Brain Image Segmentation*”. Their pipeline begins with fully optimizing an ANN, followed by a quick ANN-to-SNN conversion to initialize the corresponding spiking network. Spike-based backpropagation is then employed to fine-tune the converted SNN. Experimental results show a significant advantage of the proposed scheme over both ANN-to-SNN conversion and direct SNN training solutions in terms of segmentation accuracy and training efficiency.

In “*Chip-In-Loop SNN Proxy Learning: A New Method for Efficient Training of Spiking Neural Networks*” Liu et al. introduce the Chip-In-Loop SNN Proxy Learning (CIL-SPL) method. Their approach applies proxy learning principles and use hardware devices as proxy agents. This combination allows the hardware to exhibit event-driven, asynchronous behavior, while enabling training of the synchronous SNN structure using backward loss gradients. Experiments on the N-MNIST dataset demonstrate that CIL-SPL achieves the best performance on actual hardware chips.

## 3 Discussion and future trends

As it can be seen, the range and type of applications covered demonstrate the wide potential of SNNs in being applicable and effective in different domains, and not just for event-based visual sensing and processing, which are sometimes confused, by non-experts, almost as synonymous for SNNs. Indeed, event-based cameras are currently the most popular example of event-based sensors. The reason for this is quite likely due to the fact that event-based vision sensors provide an easy to understand approach to spike generation by mimicking the human retina. In fact, the output of an event-based camera is rather straightforward to visualise and interpret. Therefore, it is encouraging to see that the research community is exploring other avenues of application of SNNs, and this should be encouraged further. More specifically, event extraction from other sensing modalities is a very interesting and welcome feature of some of the articles in this topic.

In this respect, new commercially available neuromorphic sensors are welcome. In fact, researchers have indeed proposed novel neuromorphic sensors in other modalities, as for example audio, olfactory, tactile. However, these are still pretty much confined at academic research level. Furthermore, an interesting future direction is the investigation of novel event-based sensing approaches based on conventional, i.e. non natively neuromorphic, sensors and sensing hardware architectures. For example, as Radars and Lidars already handle information in the form of pulses, it would be ideal if existing technology could be used, as is, to generate event-based data to be fed to SNNs directly, in raw format. This would avoid the need for redundant initial transformation of the sensed data into more conventional formats, which may be unnecessary in the context of SNN-based processing.

In another sense, research on SNNs can borrow ideas from DNN approaches, but it should avoid closely mimicking the development of DNNs. As while this can provide useful research directions and hints, it may also steer SNNs toward use that does not fully exploit all the potential that event-based sensing and processing have to offer. More applications which leverage key aspects of SNN should be welcome, such as the sparse nature of event-base data, and its timing and asynchronous processing aspect. Along this line of thought, these two key aspects should be investigated to devise learning methods, that mimic the human brain and its learning, not from a mere dogmatic biological plausibility aspect, but also from a more pragmatic engineering angle.

## 4 Conclusion

Spiking Neural Networks and Neuromorphic Engineering have come out from their niche and are now known to the wider academic community and to industry, and are now frequently indicated as the key technologies that can successfully achieve AI at the Edge. This is a fundamental step forward, in order to entice further research focusing on the core aspects: sensors, processing algorithms, and hardware architectures. Nonetheless, common misconceptions should be cleared, and there should be a conscious effort to use SNNs and their capabilities for what they are, to ensure that they are used as the right tool for the job, rather than just based on their similarity to other generations of neural networks.

## Author contributions

GD: Writing – original draft, Writing – review & editing. JL: Writing – review & editing. MZ: Writing – review & editing.

